# Immune-Intrinsic Myd88 Directs the Production of Antibodies With Specificity for Extracellular Matrix Components in Primary Sjögren’s Syndrome

**DOI:** 10.3389/fimmu.2021.692216

**Published:** 2021-07-26

**Authors:** Jeremy Kiripolsky, Eileen M. Kasperek, Chengsong Zhu, Quan-Zhen Li, Jia Wang, Guan Yu, Jill M. Kramer

**Affiliations:** ^1^ Department of Oral Biology, School of Dental Medicine, The University at Buffalo, State University of New York, Buffalo, NY, United States; ^2^ Department of Immunology, Microarray & Immune Phenotyping Core Facility, University of Texas Southwestern Medical Center, Dallas, TX, United States; ^3^ Department of Biostatistics, School of Public Health and Health Professions, The University at Buffalo, State University of New York, Buffalo, NY, United States; ^4^ Department of Oral Diagnostics Sciences, School of Dental Medicine, The University at Buffalo, State University of New York, Buffalo, NY, United States

**Keywords:** decorin, biglycan, autoantibodies, toll-like receptor, NOD.B10, autoimmunity

## Abstract

Primary Sjögren’s syndrome is an autoimmune disease that is predominantly seen in women. The disease is characterized by exocrine gland dysfunction in combination with serious systemic manifestations. At present, the causes of pSS are poorly understood. Pulmonary and renal inflammation are observed in pSS mice, reminiscent of a subset of pSS patients. A growing body of evidence indicates that inflammation mediated by Damage-Associated Molecular Patterns (DAMPs) contributes to autoimmunity, although this is not well-studied in pSS. Degraded extracellular matrix (ECM) constituents can serve as DAMPs by binding pattern-recognition receptors and activating Myd88-dependent signaling cascades, thereby exacerbating and perpetuating inflammatory cascades. The ECM components biglycan (Bgn) and decorin (Dcn) mediate sterile inflammation and both are implicated in autoimmunity. The objective of this study was to determine whether these ECM components and anti-ECM antibodies are altered in a pSS mouse model, and whether this is dependent on Myd88 activation in immune cells. Circulating levels of Bgn and Dcn were similar among pSS mice and controls and tissue expression studies revealed pSS mice had robust expression of both Bgn and Dcn in the salivary tissue, saliva, lung and kidney. Sera from pSS mice displayed increased levels of autoantibodies directed against ECM components when compared to healthy controls. Further studies using sera derived from conditional knockout pSS mice demonstrated that generation of these autoantibodies relies, at least in part, on Myd88 expression in the hematopoietic compartment. Thus, this study demonstrates that ECM degradation may represent a novel source of chronic B cell activation in the context of pSS.

## Introduction

Primary Sjögren’s syndrome (pSS) is a systemic autoimmune disease characterized by loss of exocrine gland function, B cell hyperactivity and kidney and lung pathoses (1, 2). The drivers of chronic inflammation in pSS are not well understood, and consequently targeted therapies that block specific signaling cascades are not available. Damage-Associated Molecular Patterns (DAMPs) represent a potent endogenous source of inflammation that drive autoimmunity *via* activation of pattern recognition receptors. DAMPs are comprised of diverse groups of molecules, including heat shock proteins and extracellular matrix (ECM) components (3, 4). DAMP-induced inflammation is considered “sterile,” as it is caused by host-derived molecules that are normally sequestered from the immune system. When tissue becomes damaged, however, soluble DAMPs are released, thereby activating cognate receptors that mediate inflammation (4, 5). Several classes of receptors, including Myd88-dependent TLRs, are activated by DAMPs that are derived from the ECM, including biglycan (Bgn) and decorin (Dcn) (6–10).

Evidence in both SS mouse models and patients shows DAMPs may be released through pathologic degradation of exocrine tissue (11, 12). Indeed, extracts from SS salivary biopsy tissue showed elevated proteolysis of ECM proteins (11) and fibronectin is dysregulated in salivary tissue from SS mice and is elevated in saliva from SS patients (13, 14). Additionally, the ECM proteins Dcn and Bgn are degraded by saliva from pSS mice (15). While these studies provide compelling evidence that aberrant degradation of inflamed tissue facilitates release of soluble DAMPs in SS, further work is needed to understand the significance of DAMP-mediated inflammation in disease.

Mechanistic studies reveal that soluble ECM molecules can activate pathways that rely on the ubiquitously expressed cytosolic adapter, Myd88 by binding to pattern recognition receptors (8, 16, 17). Activation of Myd88 is central to many autoimmune diseases, as mice lacking Myd88 have attenuated pathology (18–23). In particular, B-cell intrinsic Myd88 plays a crucial role in autoimmunity, as lupus mice lacking Myd88 in B cells do not develop anti-nuclear antibodies (ANA) or rheumatoid factor (RF) formation (19). Additionally, our group has demonstrated that total and ANA-specific antibodies are diminished in pSS mice that lack Myd88 (24, 25). Thus, dysregulated Myd88 signaling in B cells plays an essential role in autoantibody production in autoimmunity, including pSS.

Given the importance of DAMPs in the activation of Myd88-dependent pathways in other autoimmune diseases, we performed studies to evaluate ECM expression and anti-ECM antibodies in the context of pSS using the well-established pSS mouse model, NOD.B10-*H2^b^* (NOD.B10). These animals display many disease characteristics that are reminiscent of the human disease, including female disease predilection, autoantibody production, exocrine dysfunction, and pulmonary and renal inflammation (26, 27). Additionally, conditional knockout mice derived from the NOD.B10 strain that lacked expression of Myd88 in the hematopoietic compartment (termed NOD.B10*^Myd88Δ^*) were employed (25).

Our results revealed levels of Bgn and Dcn were similar in the sera and saliva and submandibular gland (SMG) salivary tissue among the pSS strains examined. Since these ECM components mediate renal and pulmonary pathoses and both tissues show significant inflammation in pSS (1, 26, 28), Bgn and Dcn expression were evaluated in these tissues in our pSS models. Robust expression of both ECM components was detected in the lung and kidneys of all strains examined. Serum studies revealed that autoantibodies directed against ECM components were elevated in NOD.B10 female mice at the clinical disease stage when compared to healthy C57BL/10 (BL/10) animals. Moreover, numerous anti-ECM antibodies were decreased in NOD.B10*^Myd88Δ^* mice when compared to NOD.B10*^Myd88fl/fl^* controls including those directed again Bgn, Dcn, and Elastin (Eln). Thus, ECM constituents mediate autoantibody production in the context of pSS and immune-intrinsic Myd88-dependent pathways are crucial in establishing this repertoire specificity.

## Materials and Methods

### Mice

BL/10 (stock# 000666) and NOD.B10 (stock# 002591) mice are available from Jackson Laboratories. Generation and validation of pSS conditional knockout mice that lack Myd88 in the hematopoietic compartment, referred to as NOD.B10*^Myd88Δ^*, were described previously (25). Briefly, we first generated NOD.B10 mice that expressed Cre recombinase under the control of the Vav promoter (B6-Tg(vav1-icre)A2Kio/J) (Jackson Labs stock #008610) (29, 30). We then bred Myd88 floxed animals (B6.129P2(SJL)-*Myd88^tm1Defr^*/J) (Jackson Labs stock # 008888) to the NOD.B10 strain (Jackson Labs stock #002591) (31) to generate NOD.B10*^Myd88fl/fl^* mice. Animals were backcrossed to the NOD.B10 strain for at least 6 generations and were verified to be fully congenic using a speed congenics approach (Jackson Laboratories). We then bred NOD.B10^Cre-Vav^ animals to the NOD.B10*^Myd88fl/fl^* strain and the resultant progeny that expressed the Cre transgene under the control of the Vav promoter were designated as NOD.B10*^Myd88Δ^*. Littermates that did not express the Cre transgene (NOD.B10*^Myd88fl/fl^*) were employed as controls (25).

All animals used were females that were at least 26 weeks of age, the time at which the animals develop clinical disease (26, 27). All animal experiments were carried out in accordance with IACUC and NIH guidelines.

### Sera and Saliva Collection

Blood was collected by cardiac puncture immediately following euthanasia and incubated at room temperature for two hours and centrifuged at 1,300 g for 20 minutes. Sera were harvested and stored at -20°C until use. Saliva was collected following pilocarpine administration as previously described (25). Saliva was placed on ice immediately and total protein levels quantified. Saliva was solubilized using Laemmli buffer prior to storage at -20°C.

### Autoantigen Arrays

Sera were collected from NOD.B10*^Myd88Δ^* (n = 5) and NOD.B10*^Myd88fl/fl^* females (n = 5) for autoantigen arrays. Autoantibody reactivities against a panel of autoantigens were measured using an autoantigen microarray platform developed by University of Texas Southwestern Medical Center. Genepix Pro 7.0 software was used to analyze the images and generate the genepix report (GPR) files (Molecular Devices). Data were acquired and normalized as previously described (25).

### ELISAs

ELISAs were developed to detect ECM autoantibodies as follows: Absorbent plates were coated with either murine Bgn (2.5 μg/mL), murine Dcn (2.5 μg/mL) (R&D Systems) or murine Eln (2.5 μg/mL) (Millipore Sigma) and incubated overnight at 4°C. Plates were then washed with TBS containing 0.05% Tween and incubated with a blocking solution consisting of TBS with 1.5% FBS for 1 hour. Sera were serially diluted and incubated for 2 hours. Plates were washed and incubated with IgG or IgM HRP at a concentration of 1:100,000 and 1:75,000, respectively (Bethyl Laboratories). Plates were washed and autoantibodies were visualized using TMB substrate following addition of stop solution (1 N H_2_SO_4_). O.D. values were acquired at 450 nm. Serial dilutions of rabbit anti-Bgn antibody (Lifespan Biosciences, polyclonal), rat anti-Dcn antibody (R&D Systems, clone #161026) or mouse anti-Eln IgG antibody (Lifespan Biosciences, clone 10B8) with reactivity for mouse Bgn, Dcn and Eln, respectively, were used to generate standard curves to allow for normalization of values across plates.

To detect levels of Bgn and Dcn in sera, commercially available ELISAs from Lifespan Biosciences and R&D Systems, respectively, were used. Sera were diluted 1:10 (Bgn) or 1:50 (Dcn) and ELISAs were carried out in accordance with manufacturer instructions.

### RNA Isolation

RNA isolation was performed as previously described (25). Briefly, tissue was snap frozen and RNA isolated using a Qiagen RNeasy kit. cDNA synthesis was performed using an iScript kit (BioRad) and quantitative PCR (qPCR) was done with SYBR green (32). Primers used were as follows: *Bgn*: Forward: 5’-CCATCCAGGCATGTGTTCCT-3”, Reverse: 5’- GCCAGGTTGTAGCTGGGATT-3’, *Dcn*: Forward: 5’-TCGAGTGGTGCAGTGTTCT-3, Reverse: 5’-TAGCAAGGTTGTGTCGGGTG-3’, and β-Actin: Forward: 5’-TGTTACCAACTGGGACGACA-3’, Reverse: 5’-GGGGTGTTGAAGGTCTCAAA -3’.

### Immunoblots

Saliva, lung, and kidney were harvested from NOD.B10, BL/10, NOD.B10*^Myd88Δ^*, and NOD.B10*^Myd88fl/fl^* females. Western blotting was performed as previously described (25). Membranes were blotted overnight at 4°C with antibodies directed against Bgn (Lifespan Biosciences, polyclonal), Dcn (R&D Systems, clone #161026) and Vinculin (Cell Signaling Technology, clone #E1E9V). Membranes were incubated with HRP-conjugated secondary antibodies at RT for 1 hour and developed using ECL reagents (BioRad Laboratories). Tissue expression of Bgn and Dcn in lung and kidney was normalized to the housekeeping protein Vinculin using Image Lab software (BioRad Laboratories). For the saliva samples, total protein concentration in the samples was quantified by BioRad Protein Assay (BioRad Laboratories) and 10 μg of total protein was loaded for each sample.

### Statistical Analyses

Autoantigen array data were analyzed using previously described methods (26). Briefly, the two-sample t-test for all autoantigens was performed, and then the p.adjust R function in the stats R package was used to adjust the p-values for multiple comparisons. The method proposed by Benjamini and Yekutieli was used in the adjustments (33). An autoantigen was deemed significant if the corresponding adjusted p-value was less than 0.05. The autoantigen array data is deposited in the Gene Expression Omnibus (GEO) database under the accession number GSE163395. All analyses were performed using the R software. All other data were analyzed using the Mann-Whitney test with Prism software (GraphPad).

## Results

### Circulating Dcn Is Decreased in the Sera of pSS Mice

**Figure 1:**
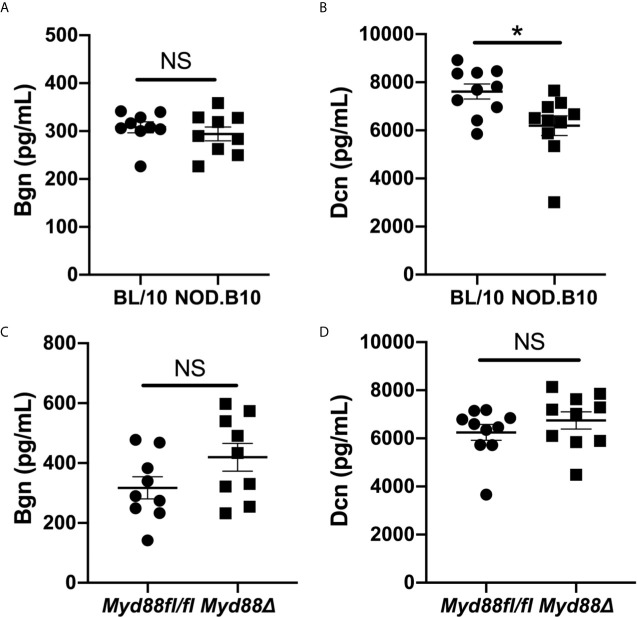
Bgn and Dcn are detected in the sera of pSS mice and controls. Sera were harvested from NOD.B10 and BL/10 mice. ELISAs were performed for **(A)** Bgn and **(B)** Dcn. Sera were also collected from NOD.B10*^Myd88fl/fl^* and NOD.B10*^Myd88Δ^* mice and ELISAs were performed for **(C)** Bgn and **(D)** Dcn. Sera from 9 or 10 mice from each strain were used for Bgn and Dcn ELISAs, respectively. Horizontal lines represent the mean and SEM, (NS, non-significant; *p < 0.05).

Since soluble Bgn and Dcn are potent inflammatory modulators that activate Myd88-dependent TLRs in the context of autoimmunity (17), we sought to determine whether circulating Bgn and Dcn were altered in pSS. We assessed sera from Myd88-sufficient pSS mice and also from those that lacked Myd88 in the hematopoietic compartment. ELISA results revealed that Bgn levels were similar in the sera of NOD.B10 females when compared to age and sex-matched controls (p = 0.5). Moreover, circulating Bgn levels remained unchanged in the NOD.B10*^Myd88Δ^* strain when compared to the NOD.B10*^Myd88fl/fl^* strain (p = 0.2) (**Figures 1A, C**). Further experiments were carried out to quantify Dcn levels in the sera of these strains. Dcn was decreased in sera derived from NOD.B10 mice (p = 0.01), although NOD.B10*^Myd88Δ^* mice had similar Dcn levels when compared to floxed controls (p = 0.2) (**Figures 1B, D**).

### Dcn and Bgn Are Detected in Salivary Tissue and Saliva From pSS Mice

**Figure 2:**
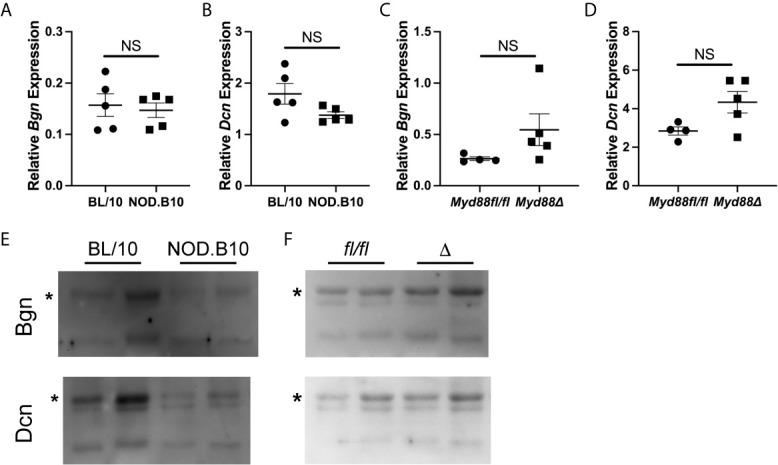
Dcn and Bgn are detected in SMG tissue and saliva. Expression of *Bgn* and *Dcn* was determined by qPCR in SMG tissue from **(A, B)** BL/10 and NOD.B10 mice (n = 5 each) and from **(C, D)** NOD.B10*^Myd88fl/fl^* and NOD.B10*^Myd88Δ^* mice (n = 4 and 5, respectively) by qPCR. Expression was normalized to β-Actin. Horizontal lines represent the mean and SEM, (NS, non-significant). Expression of Dcn and Bgn in saliva was quantified in **(E)** BL/10 and NOD.B10 mice (n = 8 and 7, respectively) and **(F)** NOD.B10*^Myd88fl/fl^* and NOD.B10*^Myd88Δ^* females (n = 4 and 5, respectively) by western blotting. Two representative animals from each strain are shown. Full-length Bgn and Dcn are indicated by asterisks. *fl/fl* = NOD.B10*^Myd88fl/fl^* and *Δ* = NOD.B10*^Myd88Δ^*.

Since salivary inflammation is a hallmark of SS and autoantibody generation is known to occur within salivary tissue in disease (34), we assessed *Bgn* and *Dcn* expression in SMG tissue derived from BL/10 and NOD.B10 females by qPCR (**Figures 2A, B**). Analagous experiments were performed in SMG tissue derived from the NOD.B10*^Myd88fl/fl^* and NOD.B10*^Myd88Δ^* strains (**Figures 2C, D**). We found *Dcn* and *Bgn* were expressed in SMG tissue, although no differences were noted among the BL/10 and NOD. B10 mice (p > 0.99 and p = 0.2, respectively) or the NOD.B10*^Myd88fl/fl^* and NOD.B10*^Myd88Δ^* strains (p = 0.06 and p = 0.1, respectively). We then sought to examine expression of Dcn and Bgn in saliva. While Dcn and Bgn were detected in the saliva of all strains, levels were similar across each of the strains (**Figures 2E, F**).

### Bgn and Dcn Are Expressed in Lung and Kidney

**Figure 3:**
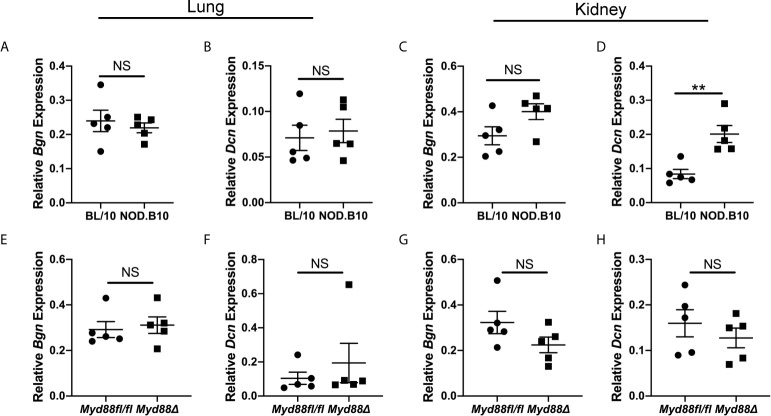
*Dcn* is elevated in the kidneys of pSS mice. Expression of *Bgn* and *Dcn* was determined in lung and kidney tissue from **(A–D)** BL/10 and NOD.B10 mice (n = 5 each) and from **(E–H)** NOD.B10*^Myd88fl/fl^* and NOD.B10*^Myd88Δ^* mice (n = 5 each) by qPCR. Expression was normalized to *β-Actin*. Horizontal lines represent the mean and SEM, (NS, non-significant; **p < 0.01).

Experiments were then carried out to identify potential tissue sources of soluble Bgn and Dcn in disease. Both ECM constituents are well-established modulators of inflammation in the lung and kidney (17, 28). While a subset of pSS patients display pulmonary and renal disease manifestations (1), the contribution of these tissues to disease pathogenesis remains poorly understood. Since NOD.B10 and NOD.B10*^Myd88fl/fl^* mice display robust kidney and renal inflammation and this is altered in NOD.B10*^Myd88Δ^* mice (25), studies were undertaken to evaluate *Bgn* and *Dcn* expression in these organs in the context of pSS (**Figure 3**). In lung tissue, levels of *Bgn* and *Dcn* were similar between NOD.B10 mice and healthy controls, and no differences were observed in *Bgn* and *Dcn* expression in lung tissue derived from the NOD.B10*^Myd88Δ^* and NOD.B10*^Myd88fl/fl^* strains (**Figures 3A, B, E, F**). Gene expression results also showed similar levels of *Bgn* and *Dcn* in kidney tissue among the strains, with the exception of elevated *Dcn* levels in the kidney of NOD.B10 mice when compared to BL/10 controls (p = 0.008) (**Figure 3D**).

**Figure 4:**
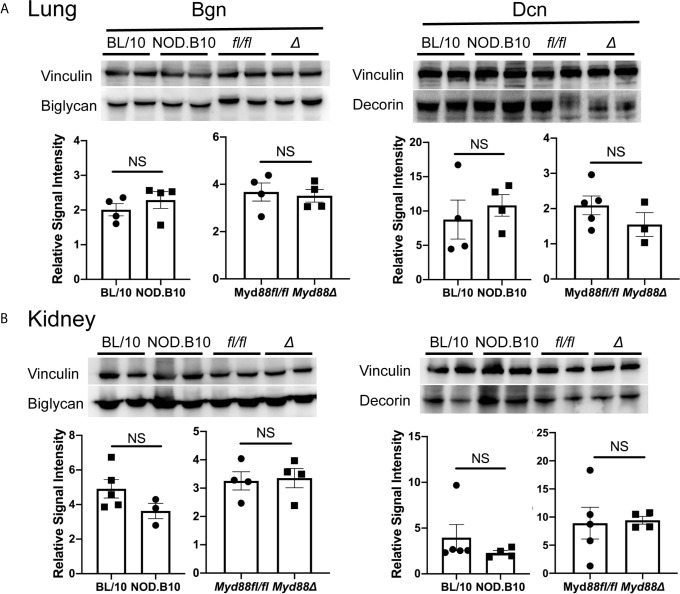
Bgn and Dcn are expressed in lung and kidney. Western blotting was performed on **(A)** lung and **(B)** kidney tissue derived from BL/10 (n = 9 or 10), NOD.B10 (n = 6 or 7), NOD.B10*^Myd88fl/fl^* (n = 8), and NOD.B10*^Myd88Δ^* mice (n = 7 or 8). Representative levels of Bgn and Dcn from 2 animals of each strain are shown and protein expression was normalized to vinculin. Data from one of two independent experiments are shown. Horizontal lines represent the mean and SEM, (NS, non-significant). *fl/fl* = NOD.B10*^Myd88fl/fl^* and *Δ* = NOD.B10*^Myd88Δ^*.

Finally, western blots were performed to evaluate expression of Bgn and Dcn (**Figure 4**). We detected Bgn in kidney and lung derived from NOD.B10 mice and BL/10 controls, as well as in the NOD.B10*^Myd88Δ^* and NOD.B10*^Myd88fl/fl^* strains, although there were no differences detected between the strains (**Figure 4**, left panels). Similarly, robust expression of Dcn was observed in lung and kidney and levels were similar across all strains examined (**Figure 4**, right panels). Thus, Bgn and Dcn are expressed in lung and kidney, and tissue-specific expression is not altered by the absence of Myd88 in the hematopoietic compartment in the context of pSS.

### Serum Autoantibodies to ECM Components Are Increased in pSS

**Figure 5:**
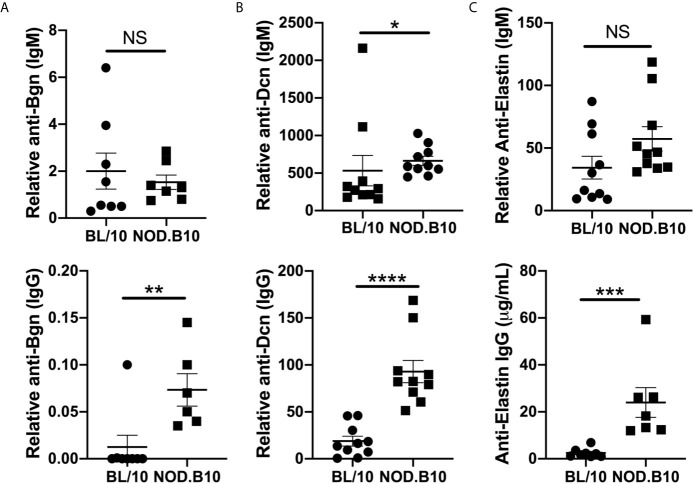
NOD.B10 females exhibit elevated autoantibodies against ECM constituents. Sera were harvested from NOD.B10 and BL/10 mice (n = at least 7 each). ELISAs were performed for IgM and IgG autoantibodies directed against **(A)** Bgn, **(B)** Dcn and **(C)** Eln. Horizontal lines represent the mean and SEM, (NS, non-significant; *p < 0.05, **p < 0.01, ***p < 0.001, ****p < 0.0001).

Previous autoantigen array studies by our group found that autoantibodies directed against ECM components were elevated in pSS mice as compared to healthy BL/10 controls (26). To confirm and extend this work, sera from NOD.B10 and BL/10 females were evaluated by ELISA for reactivity to Bgn, Dcn, and Eln (**Figure 5**). IgM autoantibodies directed against Dcn were elevated in NOD.B10 mice when compared to healthy BL/10 controls (p = 0.02) (**Figure 5B**), although no differences in IgM antibodies with specificity for Bgn or Eln were observed between sera from NOD.B10 and BL/10 mice (p = 0.7 and 0.08, respectively) (**Figures 5A, C**). Additionally, Anti-Bgn, -Dcn and Eln IgG autoantibodies were increased in the NOD.B10 females with clinical disease as compared to BL/10 controls (p = 0.008, <0.0001, and 0.0003, respectively) (**Figures 5D–F**).

### Anti-ECM Autoantibodies Are Decreased in pSS Mice That Lack Myd88 Expression in the Hematopoietic Compartment

**Figure 6:**
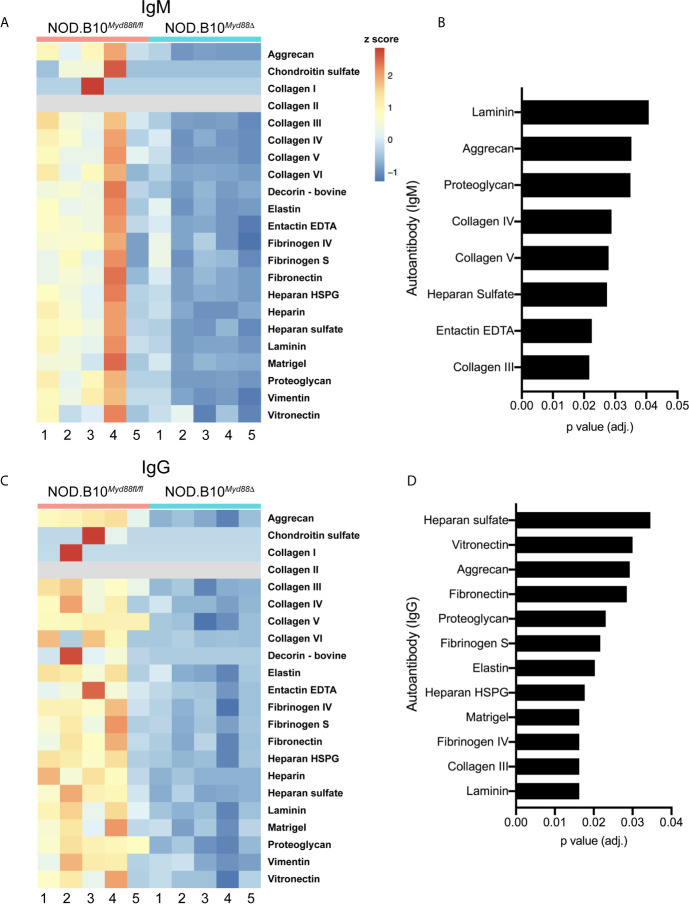
Anti-ECM antibodies are diminished in pSS animals that lack Myd88 in hematopoietic tissue. Sera were harvested from NOD.B10*^Myd88fl/fl^* and NOD.B10*^Myd88Δ^* mice (n = 5 each). **(A, C)** IgM and IgG autoantibodies were assayed by autoantigen array and heatmaps are provided to summarize the data. Analysis of autoantigen array data reveals significant differences in ECM-specific **(B)** IgM and **(D)** IgG.

Prior work by our group revealed that anti-nuclear autoantibody (ANA) production in the context of pSS relies in part on immune-intrinsic Myd88 (25). To determine if Myd88 expression in immune cells was required for generation of autoantibodies directed against ECM components, autoantigen arrays were performed on sera from pSS mice lacking Myd88 in immune cells (NOD.B10*^Myd88Δ^* mice, n = 5) and Myd88-sufficient controls (NOD.B10*^Myd88f/fl^*, n = 5) (**Figure 6**). The arrays can detect over 90 different autoantigens and we focused our analyses on the 22 autoantigens that are ECM components. Numerous IgM autoantibodies with specificity for ECM components were decreased in NOD.B10*^Myd88Δ^* mice, such as laminin, aggrecan, proteoglycan, collagen, III, collagen IV, collagen V, heparan sulfate, and entactin EDTA (**Figure 6B**). Similarly, IgG autoantibodies directed against heparan sulfate, vitronectin, aggrecan, fibronectin, proteoglycan, fibrinogen S, elastin, heparan HSPG, Matrigel, fibrinogen IV, collagen III, and laminin were diminished in NOD.B10*^Myd88Δ^* mice when compared to NOD.B10*^Myd88fl/fl^* controls (**Figure 6D**).

**Figure 7:**
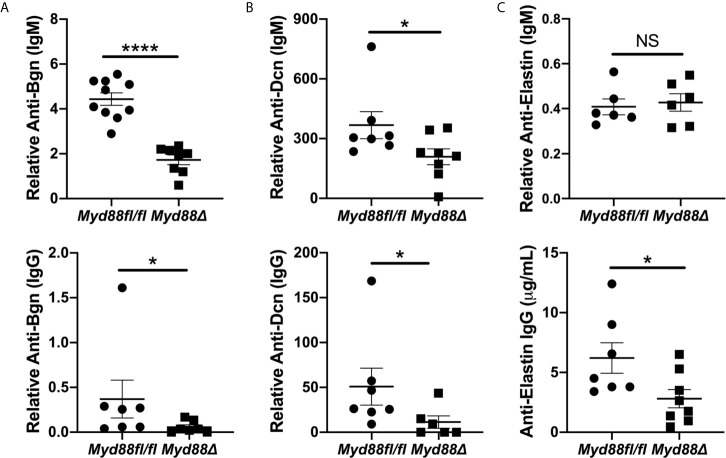
Anti-Bgn, -Dcn and -Eln autoantibodies are elevated in pSS in a Myd88-dependent manner. Sera were harvested from NOD.B10*^Myd88fl/fl^* and NOD.B10*^Myd88Δ^* mice (n = at least 7 each) and ELISAs were performed for IgM and IgG autoantibodies directed against **(A)** Bgn, **(B)** Dcn and **(C)** Eln. Horizontal lines represent the mean and SEM, (NS, non-significant; *p < 0.05, ****p < 0.00001).

To extend and validate these findings, ELISAs were performed for autoantibodies directed against Bgn, Dcn and Eln using sera from NOD.B10*^Myd88Δ^* and NOD.B10*^Myd88fl/fl^* mice (**Figure 7**). Anti-Bgn and anti-Dcn IgM antibody levels were diminished in NOD.B10*^Myd88Δ^* mice when compared with NOD.B10*^Myd88fl/fl^* controls (p < 0.0001 and p = 0.04, respectively) (**Figures 7A, B**). In agreement with autoantigen array results, anti-Eln IgM levels were similar between the NOD.B10*^Myd88Δ^* mice and the NOD.B10*^Myd88fl/fl^* controls (p = 0.9) (**Figure 7C**). IgG autoantibodies directed against Bgn, Dcn, and Eln were diminished in the NOD.B10*^Myd88Δ^* strain (p = 0.02, 0.03, and 0.03, respectively) (**Figure 7**). Taken together, these data reveal autoantibodies directed against ECM constituents are elevated in NOD.B10 mice and generation of autoantibodies with specificity for the ECM relies, at least in part, on the activation of Myd88-dependent pathways within the hematopoietic compartment.

## Discussion

DAMPs are important drivers of many autoimmune diseases (17), although the role of these endogenous molecules in pSS pathogenesis remains poorly understood. The current study was carried out to assess DAMP levels and anti-DAMP autoantibodies in the context of pSS and whether these were modulated by hematopoietic-intrinsic Myd88 expression. In particular, studies focused on Bgn and Dcn, 2 ECM components that serve as ligands for Myd88-dependent TLRs. Work herein revealed that Dcn and Bgn levels are similar in the SMG, lung and kidney among the strains examined. However, autoantibodies directed against Bgn and Dcn were increased in pSS mice, and anti-ECM antibodies were diminished in pSS mice that lacked Myd88 expression in immune cells. Thus, anti-DAMPs autoantibodies are altered in pSS, and expression of antibodies directed against ECM constituents relies, at least in part, on Myd88 activation in immune cells.

### Anti-ECM Antibodies May Contribute to Tissue Destruction Directly in Autoimmunity

Although there is a paucity of studies examining the role of anti-ECM antibodies in autoimmunity, the work published to date suggests these may play an important role in disease. In a study comparing sera derived from patients with rheumatoid arthritis (RA) to that from healthy donors, IgG autoantibodies directed against the ECM components thrombospondin-4, cartilage oligomeric matrix protein, and collagen type II were identified more frequently in RA patients when compared to healthy controls (35). Corroborative work examined anti-ECM antibodies in the synovial fluid of RA patients and those with osteoarthritis (OA) and found that anti-Bgn IgM and IgG antibodies were elevated in the synovium of RA patients when compared to those with OA (36).

Under physiologic conditions, Bgn interacts with many components of the ECM, including collagen type I, II, III, and elastin (37). Of direct relevance to disease pathogenesis, an *ex vivo* mechanistic study revealed that anti-Bgn antibodies may initiate disease by inducing collagen fiber decomposition (38). Through elegant transmission electron microscopy studies, the authors demonstrated that binding of an anti-Bgn antibody to the Bgn proteoglycan-core protein disrupted the interaction between Bgn and collagen fibrils. The Bgn core protein then dissociated from the collagen fibrils and the collagen fibril bundle decomposed into thin-fibrils. These collagen type II thin-fibrils were vulnerable to collagenase and gelatinase activity, thereby rendering the altered collagen matrix more fragile and readily digested by proteases (38). These findings have mechanistic importance for autoimmune disease, as the degraded ECM components could then become available to promote immune responses and could amplify the ongoing tissue inflammation.

Indeed, it is possible that anti-Bgn antibodies could contribute to tissue destruction and immune activation in pSS. Our data demonstrate that anti-Bgn IgG antibodies are elevated in NOD.B10 mice (**Figure 5A**). Moreover, both IgM and IgG antibodies directed against Bgn are decreased in NOD.B10*^Myd88Δ^* mice when compared to floxed controls (**Figure 7A**). Thus, these antibodies may facilitate collagen degradation in disease in a Myd88-dependent manner. This putative disease mechanism is supported by the autoantigen array data, as autoantibodies directed against several types of collagen are diminished in NOD.B10*^Myd88Δ^* mice (**Figure 6**).

Corroborative work demonstrates the presence of Bgn degradation products in submandibular gland lysates from NOD.B10 mice (15). Moreover, increased gelatinase activity was detected in the saliva of NOD.B10 mice with clinical disease and elevated levels of *Mmp2* and *Mmp9* were identified in salivary tissue (15, 39). These findings may have pathologic significance, as a Bgn neo-epitope generated by concomitant MMP9 and MMP12 digestion was elevated in a rat model of RA and levels of this neo-epitope correlated with liver fibrosis in a rat bile duct ligation model (40). Of note, several small molecular weight products were detected using a polyclonal Bgn antibody in our mouse strains in the saliva, lung and kidney in the current study (**Figure 2** and **Supplemental Figure 1**). It is interesting to speculate that these may represent Bgn degradation products that could carry pathogenic consequence in the context of pSS (15), although further experiments are needed to establish this conclusively.

### Soluble Dcn and Bgn Are Elevated in Autoimmunity and Modulate Inflammation

While the tissue source of soluble ECM constituents in pSS remains poorly understood, these components could activate many different signaling networks in pSS, as Bgn binds CD14, TLR2, TLR4, CD44 and the purinergic receptors P2X_7_/P2X_4_ (6, 7, 41–43). Additional studies have identified functional interactions between Dcn and TLR2 and TLR4 (32, 44). While Bgn and Dcn activate pro-inflammatory cascades, anti-inflammatory signaling outcomes are also documented (17). Of relevance to the current study, select pathways activated by Bgn require Myd88 (7, 42, 45, 46). Dcn likely activates Myd88-dependent pathways as well, since TLR2 requires Myd88 for signal transduction, although this has not been confirmed experimentally to date to our knowledge.

In pSS, Dcn induced both pro- and anti-inflammatory mediators in splenocytes (32). This finding, in conjunction with those of the current study, suggest that Dcn could induce both protective and destructive changes in the context of pSS depending on the tissue microenvironment. This dichotomous role for Myd88-mediated signaling is supported by our previous work in NOD.B10*^Myd88Δ^* mice. Interestingly, pulmonary inflammation was heightened in NOD.B10*^Myd88Δ^* mice but was diminished in the kidney when compared to floxed controls (25). Taken together, these findings highlight the need for further studies in specific tissues to delineate the way in which Bgn and Dcn modulate inflammation in pSS.

### DAMPs Mediate B Cell Activation in Autoimmunity Through Myd88-Dependent Pathways

While the significance of B cell-intrinsic Myd88-dependent TLR signaling in pSS is poorly understood, the importance of these pathways in other autoimmune diseases is evident. In lupus, a related autoimmune disease (47), B cells express TLRs and BCRs that have shared specificity for nuclear autoantigens. These antigens are released from apoptotic or necrotic cells, thereby engaging BCR and TLR signaling concomitantly to induce B cell activation (23, 48, 49). Elegant studies in lupus models revealed that recognition of endogenous nuclear antigens by B cell TLRs is necessary for autoantibody production in lupus and this facilitates the generation of ANA-secreting cells in a Myd88-dependent manner (49).

This paradigm extends beyond nuclear autoantigens, as generation of RF also relies on TLR7 and TLR9 (50). Importantly, TLR2 and TLR4 are crucial for autoantibody generation in lupus (51, 52) and a TLR4-deficient arthritis model shows attenuated disease and lower titers of autoantibodies (53). Moreover, studies in a scleroderma model demonstrate stimulation of B cells with the ECM component hyaluronan activates TLR2 and TLR4 and results in inflammatory cytokine secretion, including IL-6, TNFα, and IFNγ (54). In agreement with these findings, results from the current study suggest that recognition of soluble ECM components by BCRs together with simultaneous activation of Myd88-dependent signaling networks may represent a previously unappreciated mechanism of B cell activation in pSS.

### Bgn and Dcn Are Implicated in Diverse Pathoses Affecting the Lung and Kidney

An increasing number of studies demonstrate a role for Dcn and Bgn in autoimmunity and there is considerable evidence that DAMPs contribute to both pulmonary and renal pathology. Indeed, DAMP-mediated inflammatory networks are identified in asthma, pulmonary fibrosis, chronic obstructive pulmonary disease and in lung cancer (17, 28, 55). Similarly, DAMPs contribute to many distinct kidney pathoses, such as renal fibrosis, lupus nephritis, and diabetic nephropathy (7, 28, 56). Of relevance to the work herein, when soluble biglycan was overexpressed in healthy mice, levels of pro-inflammatory cytokines were increased in kidney lysates (7). Dcn can also stimulate production of inflammatory mediators in peritoneal macrophages, but this has not been documented in the lung or kidney to date (28, 44). Separate studies, however, reveal a protective role for Dcn in the context of disease. Indeed, in a model of streptozotocin-induced diabetes, *Dcn-/-* mice showed accelerated diabetic nephropathy (57, 58). Moreover, in lung tissue derived from *Dcn*-deficient mice with sepsis, IL-10 levels were decreased and IL-12 and TNFα levels were increased (44). Thus, further studies are warranted to understand the signaling networks activated by Bgn and Dcn in greater depth and to identify the organ-specific consequences of Myd88 activation by Dcn and Bgn in pSS.

## Conclusion

Data from the current study reveal that the ECM components Bgn and Dcn are expressed in the sera, SMG, saliva, lung, and kidney of pSS mice and controls. Autoantibodies directed against both Bgn and Dcn are elevated in pSS mice, and NOD.B10*^Myd88Δ^* mice that lack Myd88 expression in the hematopoietic compartment show diminished expression of many autoantibodies that bind ECM components, including Dcn and Bgn. Thus, these data reveal that ECM degradation products may represent a novel source of B cell activation in pSS and therapeutics that target Myd88-dependent signaling cascades may have therapeutic efficacy in the context of this disease.

## Data Availability Statement

The datasets presented in this study can be found in online repositories. The names of the repository/repositories and accession number(s) can be found below: https://www.ncbi.nlm.nih.gov/geo/, GSE163395.

## Ethics Statement

The animal study was reviewed and approved by The University at Buffalo Institutional Animal Care and Use Committee

## Author Contributions

JMK conceived of the work, wrote the manuscript, and performed experiments. JK and EK critically edited the manuscript and performed experiments. CZ and Q-ZL performed the autoantigen arrays and normalized the data. JW and GY critically edited the manuscript and analyzed the autoantigen array. All authors contributed to the article and approved the submitted version.

## Funding

Funding for this work was provided by NIH/National Institute of Dental and Craniofacial Research (NIDCR) awards R21DE027489 and R01DE29472 to JMK. Funding was also provided by the NIH/National Institute of Dental and Craniofacial Research (NIDCR) and Office of the Director, National Institutes of Health (OD) award R56DE25218. Research reported in this publication was supported by the National Center for Advancing Translational Sciences of the National Institutes of Health under award number UL1TR001412 to the University at Buffalo.

## Author Disclaimer

The content is solely the responsibility of the authors and does not necessarily represent the official views of the NIH.

## Conflict of Interest

The authors declare that the research was conducted in the absence of any commercial or financial relationships that could be construed as a potential conflict of interest.

## Publisher’s Note

All claims expressed in this article are solely those of the authors and do not necessarily represent those of their affiliated organizations, or those of the publisher, the editors and the reviewers. Any product that may be evaluated in this article, or claim that may be made by its manufacturer, is not guaranteed or endorsed by the publisher.

## Supplementary Material

Supplemental Figure 1 Bgn is expressed in lung and kidney. Western blotting was performed on **(A)** lung and **(B)** kidney tissue derived from BL/10 (n = 9 or 10), NOD.B10 (n = 6 or 7), NOD.B10Myd88fl/fl (n = 8), and NOD.B10Myd88δ mice (n = 7 or 8). Representative levels of Bgn from 2 animals of each strain are shown and protein expression was normalized to vinculin as shown in figure 4. Data from one of two independent experiments are shown. The red line indicates full-length Bgn and the black lines indicate possible Bgn degradation products. fl/fl = NOD.B10Myd88fl/fl and Myd88δ = NOD.B10Myd88δ).
