# Supplementary figures and images for: Immune-Intrinsic Myd88 Directs the Production of Antibodies With Specificity for Extracellular Matrix Components in Primary Sjögren’s Syndrome
Source: Front Immunol. 2021 Jul 26;12:692216. doi: 10.3389/fimmu.2021.692216 (PMC8350326; doi:10.3389/fimmu.2021.692216)

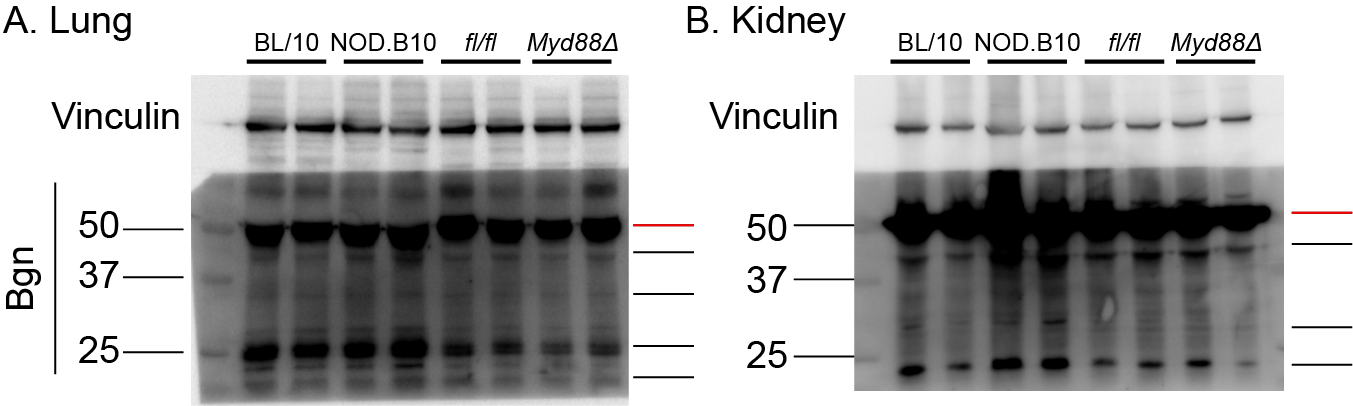

Supplement: Supplemental Figure 1 — Bgn is expressed in lung and kidney. Western blotting was performed on (A) lung and (B) kidney tissue derived from BL/10 (n = 9 or 10), NOD.B10 (n = 6 or 7), NOD.B10Myd88fl/fl (n = 8), and NOD.B10Myd88δ mice (n = 7 or 8). Representative levels of Bgn from 2 animals of each strain are shown and protein expression was normalized to vinculin as shown in figure 4. Data from one of two independent experiments are shown. The red line indicates full-length Bgn and the black lines indicate possible Bgn degradation products. fl/fl = NOD.B10Myd88fl/fl and Myd88δ = NOD.B10Myd88δ). [file Image_1.tif]
